# An Adaptive B-Spline Neural Network and Its Application in Terminal Sliding Mode Control for a Mobile Satcom Antenna Inertially Stabilized Platform

**DOI:** 10.3390/s17050978

**Published:** 2017-04-28

**Authors:** Xiaolei Zhang, Yan Zhao, Kai Guo, Gaoliang Li, Nianmao Deng

**Affiliations:** 1 School of Instrumentation Science and Opto-Electronics Engineering, Beihang University, Beijing 100191, China; zhaoyanbuaa@163.com (Y.Z.); k_guo@buaa.edu.cn (K.G.); ligaoliang@buaa.edu.cn (G.L.); 2 Beijing Institute of Control & Electronic Technology, Beijing 100038, China; zxlbuaa@126.com

**Keywords:** mobile satcom antenna, inertial sensor, stabilized platform, B-spline neural network, terminal sliding mode (TSM)

## Abstract

The mobile satcom antenna (MSA) enables a moving vehicle to communicate with a geostationary Earth orbit satellite. To realize continuous communication, the MSA should be aligned with the satellite in both sight and polarization all the time. Because of coupling effects, unknown disturbances, sensor noises and unmodeled dynamics existing in the system, the control system should have a strong adaptability. The significant features of terminal sliding mode control method are robustness and finite time convergence, but the robustness is related to the large switching control gain which is determined by uncertain issues and can lead to chattering phenomena. Neural networks can reduce the chattering and approximate nonlinear issues. In this work, a novel B-spline curve-based B-spline neural network (BSNN) is developed. The improved BSNN has the capability of shape changing and self-adaption. In addition, the output of the proposed BSNN is applied to approximate the nonlinear function in the system. The results of simulations and experiments are also compared with those of PID method, non-singularity fast terminal sliding mode (NFTSM) control and radial basis function (RBF) neural network-based NFTSM. It is shown that the proposed method has the best performance, with reliable control precision.

## 1. Introduction

A mobile satcom antenna (MSA) is a kind of satellite communication antenna which can maintain continuous and reliable communications between satellites and users in movement. MSAs are widely applied in vehicle carriers, flying platforms and vessels. The Jet Propulsion Laboratory (JPL) has developed several mobile vehicular antenna systems for the satellite-based applications [1]. Due to the uncertain working environment, such as bumpy roads, sea waves and airflows, there might be strong disturbances in actual systems [2]. MSAs seeking to achieve high data-rate communications require the inertial antenna to be capable of pointing to within fractions of a degree [3]. Stabilization is used in the antenna pointing control system to maintain the line of sight by isolating the disturbances caused by vehicle motion [4]. Generally speaking, the attitude sensor can be an attitude and heading reference system (AHRS) or an inertial measurement unit (IMU) [5]. The outputs of these inertial sensors are widely used as feedbacks for stabilization in antenna control systems [6]. The accuracy of control is influenced by many factors, mainly including the existence of unmodeled nonlinear uncertainty, random bias of states and sensor noises [7] and friction restriction, which decreases the accuracy most. The disturbances and uncertainties can lead to the degradation of tracking and stabilization accuracy of inertial stabilized platform in engineering applications [8].

Various decoupling control and disturbance rejecting methods have been developed to enable MSA with high performance. Though the PID control method is widely applied in engineering practice due to its simplicity and reliability, it is difficult to reduce the disturbances and uncertainties rapidly [8]. Therefore, this method offers low control accuracy and robustness. A feedforward scheme for a two axis inertial stabilized platform (ISP) was adopted to reject the periodic disturbing torque acting on the payload due to the static mass unbalance [9,10], but the accelerometer signal may be easily distorted by the centripetal acceleration [11]. The *H*_∞_ control method presents strong robustness, but it is highly conservative and the control accuracy is usually sacrificed [12]. Internal mode control can improve the disturbance rejection capability and robustness against model uncertainties, but the exact structural property of the disturbance needs to be known in advance [13]. The sliding mode control method is studied in depth for its unique robust control performance for nonlinear and uncertain systems. In the linear sliding mode, the deviation between the system and the desired trajectory is asymptotically convergent in exponential forms [14]. TSM control is improved and compared with the linear hyperplane based sliding modes, showing that the TSM offers some superior properties such as fast response, finite time convergence [15,16].

However, the conventional TSM has many defects such as singular problem and chattering phenomena. An initial discussion about avoiding the singularity in TSM control was presented in [17]. In order to avoid the singular problem, many non-singular TSMs were proposed [14,15,18,19,20]. The good robustness of TSM is based on the large switching control gain, which is determined by the uncertain disturbances [20]. If the switching control gain is small, the uncertainty issues cannot be eliminated, whereas if the gain is large enough, the chattering phenomena would be enhanced. In many practical control systems, including DC motors and aircraft control system, it is important to avoid control chattering by providing continuous or smooth control signals [21]. According to the high order sliding mode algorithm proposed in [22], “chattering removal” can be achieved by combining the arbitrary-order sliding mode controller with the dynamic sliding mode. Another well-known way to reduce chattering problem in TSM is to use tanh function instead of the sign function [23]. Neural networks can learn and approximate any arbitrary nonlinear functions [24]. Therefore, neural networks are implemented to approximate uncertain disturbances in systems as well as to reduce the chattering [25].

A radial basis function (RBF) neural network has several important features such as simple structure, fast learning and better approximation capabilities. It has been adopted in TSM control systems [24,26]. Wavelet neural networks, which are applied to terminal sliding mode control [27,28], are a new class of neural networks that have been developed using a combined method of multi-layer artificial neural networks and wavelet analysis. The B-spline function is a piecewise polynomial function widely used in computer-aided design and computer graphics. For its excellent local features, the B-spline function can be used as an activation function of neural network. The B-spline neural networks (BSNNs) can be divided into two groups: one group is based on the B-spline basis function [29,30,31,32] and the other group is based on the B-spline curve [33,34,35,36,37,38].

The B-spline basis function is only related to the internal knots, hence the definite internal knots can derive the definite B-spline basis curve. For those kinds of BSNNs, only the weights are trained in some studies [29,31]. The internal knots are trained by a heuristic algorithm in some other studies [30,32], making the shapes of B-spline basis function changeable. In this group, the input of the neural network can be considered as the value of internal knots, then according to the b-Spline basis function, the output of the neural network can be obtained.

In the group where the B-spline curve is adopted, it is known that the B-spline curve is a linear combination of control points and B-spline basis functions. If the positions of the control points are changed, the shapes of B-spline curves are also changed. In some studies, the weights are considered as control points [38]. Through the training of weights, the BSNN can then become adaptive. In other references, control points are trained by rules. In [33,37], the position of control points can be changed to a known nearby value, but only one control point can be changed once by this method. Additionally, the input and output of neural network are not relevant directly in this group.

In this paper, a novel B-spline neural network with the capability of shape adjustment is proposed and applied to approximate the external disturbance and the unmolded dynamics of the system. The equation of the B-spline curve is reconstructed in numeric form, therefore the new B-spline curve possesses the properties of the radial basis function, making it different from the previous approaches. In this research, the activation function of proposed BSNN is a pp-form spline.

The paper is organized as follows: in Section 2, the dynamic model of MSA and the control block diagram are established respectively. Then a new terminal sliding mode control strategy is proposed and proved. In Section 3, a novel BSNN is proposed and the parameter updating rules are built. In Section 4, experiments and simulations are carried out. The performances of the proposed methods are also compared with other methods. Finally, conclusions and contributions are summarized in Section 5.

## 2. Modeling of a Mobile Satcom Antenna

The paper focuses on a double gimbal MSA. The innermost gimbal is an elevation gimbal on which the antenna array is mounted, while the outmost gimbal is an azimuth gimbal which is mounted on the base. According to the coordinates system definition and the conversion relationships between different coordinates, a dynamic model of the mobile satcom antenna is derived.

### 2.1. Gimbal Coordinates Definition

**Figure 1:**
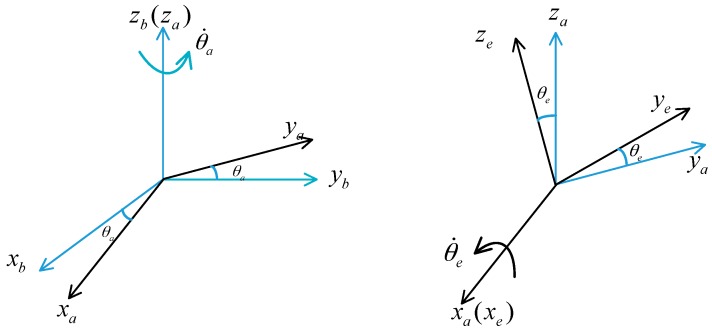
Coordinate definition and transformation.

An orthogonal coordinate system is defined: Base coordinate frame $\left(ox_{b}y_{b}z_{b}\right)$, azimuth gimbal frame $\left(ox_{a}y_{a}z_{a}\right)$ and elevation gimbal frame $\left(ox_{e}y_{e}z_{e}\right)$. The rotating transformations are shown in Figure 1. As we can see from the Figure 1, $\theta_{a}$ and ${\dot{\theta }}_{a}$ are the relative angular and relative angular rate between the azimuth gimbal and the base carrier, respectively. $\theta_{e}$ and ${\dot{\theta }}_{e}$ are the relative angular and relative angular rate between the elevation gimbal and the azimuth gimbal, respectively:

Rotation matrices can be written as $C_{b}^{a}=\left[\begin{array}{ccc} \cos\theta_{a} & \sin\theta_{a} & 0\\ -\sin\theta_{a} & \cos\theta_{a} & 0\\ 0 & 1 & 1 \end{array}\right]$ and $C_{a}^{e}=\left[\begin{array}{ccc} 1 & 0 & 0\\ 0 & \cos\theta_{e} & \sin\theta_{e}\\ 0 & -\sin\theta_{e} & \cos\theta_{e} \end{array}\right]$, which represent the transformation from the base coordinate to the azimuth coordinate and transformation from azimuth coordinate to the elevation coordinate, respectively.

According to the rotation matrix $C_{b}^{a}$ and $C_{a}^{e}$, the angular rate relations of the two gimbals can be obtained as follows:

(1) $$\omega_{ia}^{a}=C_{b}^{a}\omega_{ib}^{b}+\omega_{ba}^{a}=\left[\begin{array}{c} p\cos\theta_{a}+q\sin\theta_{a}\\ -p\sin\theta_{a}+q\cos\theta_{a}\\ r+{\dot{\theta }}_{a} \end{array}\right]$$

(2) $$\omega_{ie}^{e}=C_{a}^{e}\omega_{ia}^{a}+\omega_{ae}^{e}=\left[\begin{array}{c} p\cos\theta_{a}+q\sin\theta_{a}+{\dot{\theta }}_{e}\\ -p\cos\theta_{e}\sin\theta_{a}+q\cos\theta_{a}\cos\theta_{e}+r\sin\theta_{e}+{\dot{\theta }}_{a}\sin\theta_{e}\\ p\sin\theta_{a}\sin\theta_{e}-q\cos\theta_{a}\sin\theta_{e}+r\cos\theta_{e}+{\dot{\theta }}_{a}\cos\theta_{e} \end{array}\right]$$

in which, $\omega_{ib}^{b}$ is the angular rate of the base carrier; $\omega_{ia}^{a}$ is the angular rate of the azimuth gimbal; $\omega_{ie}^{e}$ is the angular rate of the elevation gimbal; $\omega_{ba}^{a}={\left[\begin{array}{ccc} 0 & 0 & {\dot{\theta }}_{a} \end{array}\right]}^{T}$ represents the angular rate of the azimuth gimbal with respect to the base; $\omega_{ae}^{e}={\left[\begin{array}{ccc} {\dot{\theta }}_{e} & 0 & 0 \end{array}\right]}^{T}$ represents the angular rate of the elevation gimbal with respect to the azimuth gimbal; $\omega_{ib}^{b}={\left[\begin{array}{ccc} p & q & r \end{array}\right]}^{T}$ is the angular rate of the mobile carrier.

### 2.2. Dynamic of the Azimuth Gimbal

The gimbals in this system are all rigid, therefore the basic Newton-Euler rotation equation can be written as follows:

(3) $$\begin{array}{l} \sum M=\dot{H}+\omega \times H\\ H=J\omega \end{array}$$

in which ∑M is the resultant moment of the force added to the rigid body; H is the inertial angular momentum; J is the inertial moment; ω is the absolute angular rate.

Since the structures of the two gimbals are similar (the difference is that the azimuth gimbal is effected by coupling of elevation gimbal), only the azimuth gimbal is considered in this paper.

According to the Equation (3), the dynamic model of azimuth gimbal can be obtained as follows:

(4) $$\left(J_{az}+J_{ez}\right)(\dot{r}+{\ddot{\theta }}_{a})=M_{am}-M_{ad}-M_{aez}-(J_{ay}+J_{ey}-J_{ax}-J_{ex})\omega_{iax}^{a}\omega_{iay}^{a}$$

where, $M_{ad}$ is the disturbance torque added to elevation gimbal; $M_{am}$ is the driving torque of elevation motor; $M_{aez}$ is the coupling torque that effected by elevation gimbal. They are given by Equations (5) and (6):

(5) $$M_{aez}=J_{ez}{\dot{\omega }}_{iez}^{e}\sin\theta_{e}+\left(J_{ex}-J_{ey}\right)\omega_{iex}^{e}\omega_{iey}^{e}\sin\theta_{e}+J_{ez}{\dot{\omega }}_{iez}^{e}\cos\theta_{e}+\left(J_{ex}-J_{ey}\right)\omega_{iex}^{e}\omega_{iey}^{e}\cos\theta_{e}$$

(6) $$M_{am}=\frac{k_{at}}{R_{aa}}\left(N_{a}u_{aa}-N_{a}^{2}k_{ab}{\dot{\theta }}_{a}\right)-\left(N_{a}^{2}J_{ad}+J_{af}\right){\ddot{\theta }}_{a}-\left(N_{a}^{2}f_{ad}+f_{af}\right){\dot{\theta }}_{a}$$

$J_{ax},J_{ay},J_{az}$ are the moments of inertia of the azimuth gimbal related to the X,Y,Z axes in azimuth coordinates, respectively; $J_{ex},J_{ey},J_{ez}$ are the moments of inertia of the elevation gimbal related to the X,Y,Z axes in the elevation gimbal, respectively; $k_{at}$ is the azimuth motor torque constant; $k_{ab}$ is the back-EMF coefficient of azimuth motor; $N_{a}$ is the azimuth gear ratio; $R_{aa}$ is the motor resistance; $J_{ad}$ is the moment of inertial of driver gear; $f_{ad}$ is the viscous friction coefficient of driver gear; $J_{af}$ is the moment of inertial of passive gear; $f_{af}$ is the viscous friction coefficient of passive gear; $u_{aa}$ is the input driven voltage of the motor.

Based on the preceding analysis, system (4) can be simplified into a standard second order control system:

(7) $$\{\begin{array}{c} {\dot{x}}_{a1}\left(t\right)=x_{a2}\left(t\right)\\ {\dot{x}}_{a2}(t)=A_{a}x_{a2}(t)+B_{a}u_{a}(t)+C_{a}d_{a}(t)+D_{a}w_{a}(t) \end{array}$$

in which, $x_{a1}=\theta_{a}$, $x_{a2}={\dot{\theta }}_{a}$, $d_{a}\left(t\right)$ is external disturbance and $w_{a}\left(t\right)$ is the coupling effect torque. To further simplify the analysis, we define that $\left|C_{a}d_{a}\left(t\right)+D_{a}w_{a}\left(t\right)\right|\leq l_{d}$. Then the proposed control block diagram can be obtained as follows:

$$A_{a}=\left[\begin{array}{cc} 0 & 1\\ 0 & -\frac{k_{at}k_{ab}N_{a}^{2}+R_{aa}N_{a}^{2}f_{ad}+R_{aa}f_{af}}{(J_{az}+J_{ez}+N_{a}^{2}J_{ad}+J_{af})R_{aa}} \end{array}\right],\;B_{a}=\left[\begin{array}{c} 0\\ \frac{k_{at}N_{a}}{(J_{az}+J_{ez}+N_{a}^{2}J_{ad}+J_{af})R_{aa}} \end{array}\right]$$

$$C_{a}=\left[\begin{array}{c} 0\\ -\frac{1}{J_{az}+J_{ez}+N_{a}^{2}J_{ad}+J_{af}} \end{array}\right],\;D_{a}=\left[\begin{array}{c} 0\\ -\frac{1}{J_{az}+J_{ez}+N_{a}^{2}J_{ad}+J_{af}} \end{array}\right]$$

and $d_{a}\left(t\right)=M_{ad}+\left(J_{az}+J_{ez}\right)\dot{r}+\left(J_{ay}+J_{ey}-J_{ax}-J_{ex}\right)\omega_{iax}^{a}\omega_{iay}^{a}$.

**Figure 2:**
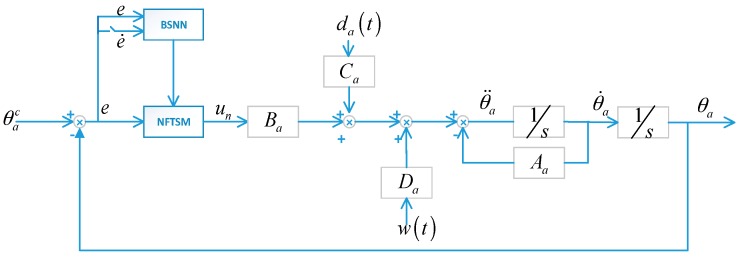
Control block diagram of azimuth gimbal.

Then the control block diagram shown in Figure 2 can be obtained.

The NFTSM control applied in this paper can guarantee that the system state arrives at the equilibrium point in a finite time with fast response and higher precision. In the following section, a new NFTSM controller is proposed and proved.

### 2.3. Non-Singularity Fast Terminal Sliding Mode

The nonlinear system is defined by Equation (7). In order to improve the control performance of terminal sliding mode, the paper proposed an improved NFTSM as shown in Equation (8), which is similar to reference [14].

(8) $$s=\hat{e}+\frac{1}{\alpha }{\left\|\hat{e}\right\|}^{r+1}sign\left(\hat{e}\right)+\frac{1}{\beta }{\left\|\dot{\hat{e}}\right\|}^{p+1}sign\left(\dot{\hat{e}}\right)=0$$

in which, α,β>0, 0<p<1 and r>p, $\hat{e}=\theta_{a}-\theta_{a}^{c}$. The first derivative can be expressed as

(9) $$\dot{s}=\dot{\hat{e}}+\frac{r+1}{\alpha }{\left\|\hat{e}\right\|}^{r}\dot{\hat{e}}+\frac{p+1}{\beta }{\left\|\dot{\hat{e}}\right\|}^{p}\ddot{\hat{e}}$$

**Theorem** **1.** *For System (7) with the adopted NFTSM, if the control law is designed as:*

(10) $$u_{n}=-{B_{a}}^{-1}\left[\begin{array}{l} \frac{\beta }{p+1}{\left\|\dot{\hat{e}}\right\|}^{1-p}sign\left(\dot{\hat{e}}\right)\left(1+\frac{r+1}{\alpha }{\left\|\hat{e}\right\|}^{r}\right)\\ +A_{a}\dot{\hat{e}}+\left(l_{d}+\delta \right)sign\left(s\right)+\left(A_{a}{\dot{\theta }}_{a}^{c}-{\ddot{\theta }}_{a}^{c}\right) \end{array}\right]$$

*the NFTSM manifold will be reached in a finite time. In addition, the tracking error on the sliding mode surface will also converge to zero in a finite time.*

**Proof.**  Consider the Lyapunov candidate function as:

(11) $$V=\frac{1}{2}s^{2}$$

The derivative of V is given by the following function,

(12) $$\begin{array}{ll} \dot{V} & =s\dot{s}=s\left(\begin{array}{l} \dot{\hat{e}}+\frac{r+1}{\alpha }{\left\|\hat{e}\right\|}^{r}\dot{\hat{e}}+\frac{p+1}{\beta }{\left\|\dot{\hat{e}}\right\|}^{p}A_{a}\dot{\hat{e}}+\frac{p+1}{\beta }{\left\|\dot{\hat{e}}\right\|}^{p}B_{a}u_{n}\left(t\right)+\\ \frac{p+1}{\beta }{\left\|\dot{\hat{e}}\right\|}^{p}C_{a}d_{n}\left(t\right)+\frac{p+1}{\beta }{\left\|\dot{\hat{e}}\right\|}^{p}D_{a}w_{n}\left(t\right)+\frac{p+1}{\beta }{\left\|\dot{\hat{e}}\right\|}^{p}\left(A_{a}{\dot{\theta }}_{a}^{c}-{\ddot{\theta }}_{a}^{c}\right) \end{array}\right)\\ & =s\left(-\frac{p+1}{\beta }{\left\|\dot{\hat{e}}\right\|}^{p}\left(\left(l_{d}+\delta \right)sign\left(s\right)\right)+\frac{p+1}{\beta }{\left\|\dot{\hat{e}}\right\|}^{p}C_{a}d_{n}\left(t\right)+\frac{p+1}{\beta }{\left\|\dot{\hat{e}}\right\|}^{p}D_{a}w_{n}\left(t\right)\right)\\ & \leq -s\left(\delta sign\left(s\right)\right)=-\left|s\right|\delta \end{array}$$

It is obvious that $\dot{V}<0$ if $\dot{\sigma }=0$. Thus, the condition for Lyapunov stability is satisfied. Then the system state will converge to zero within finite time. As shown in Equation (10), the value of 1−p<0 is always greater than zero, therefore, the controller is non-singular. ☐

We can also obtain the reaching time $t_{r}$ satisfies (see Appendix A)

(13) tr≤p+1pβ1p+1|e(t0)|pp+1

That completes the proof. Therefore, the system can reach the terminal sliding mode surface within a finite time.

For terminal sliding mode control methods, the upper disturbance bound is generally required, but in practical applications, a large upper bound can be used to eliminate the external disturbance item and guarantee the robustness. However, the chattering phenomena is enhanced correspondingly. The paper attempts to approximate nonlinear disturbance by using BSNN. A novel BSNN is proposed in this paper. As its name implies, the B-spline curve is adopted as activation function.

## 3. B-Spline Neural Network

Artificial neural networks have high nonlinear approximation abilities. A spline function is a piecewise polynomial function of degree k. The spline neural network can play a role in local features. In this section, a new kind of B-spline function is introduced herin, then the proposed BSNN is deduced.

### 3.1. B-Spline Basis Function Definition

A B-spline curve differs from a Hermite or Bérzier curve, because a B-spline curve usually consists of more than one curve segment. The B-spline curve is widely used in the field of computer graphics for its excellent performance. The B-spline basis function is the foundation of BSNN.

**Definition** **1** [39]**.** *Given a knot vector* $T=\left\{t_{0}\leq t_{1}\leq \cdots \leq t_{m}\right\}$*, the* ith *B-spline basis function of degree k can be written as:*

(14) $$B_{i,0}(t)=\{\begin{array}{l} 1,\;if\;t_{i}<=t<t_{i+1}\\ 0,otherwise \end{array}$$

(15) $$B_{i,k}\left(t\right)=\frac{t-t_{i}}{t_{i+k-1}-t_{i}}\cdot B_{i,k-1}\left(t\right)+\frac{t_{i+k}-t}{t_{i+k}-t_{i+1}}\cdot B_{i+1,k-1}\left(t\right)$$

**Remark** **1.** *If* $t_{i+1}-t_{i}\left(0\leq i\leq m-1\right)$ *is a constant, the B-spline curve is called uniform; otherwise, it is called non-uniform.*

Definition 1 is usually referred to the Cox-de Boor recursion formula.

The jth B-spline basis curve with degree *k* can be written as $B_{j,k}^{i}\left(t\right)$ on an arbitrary vector $[t_{i},t_{i+1})$:

(16) $$B_{j,k}^{i}\left(t\right)=a_{j,0}^{i}+a_{j,1}^{i}t+a_{j,2}^{i}t^{2}+\cdots +a_{j,k}^{i}t^{k}=\left[\begin{array}{cccc} t^{k} & t^{k-1} & \cdots & 1 \end{array}\right]\left[\begin{array}{c} a_{j,k}^{i}\\ \vdots \\ a_{j,1}^{i}\\ a_{j,0}^{i} \end{array}\right]$$

An order k+1 B-spline is formed by joining several pieces of polynomials of degree *k* with at most $C^{k-1}$ continuity at the breakpoints [40], hence the following formula can be obtained:

(17) $$\left[\begin{array}{ccccc} t_{i}{}^{k} & t_{i}{}^{k-1} & \cdots & t_{i} & 1\\ t_{i+1}{}^{k} & t_{i+1}{}^{k-1} & \cdots & t_{i} & 1\\ kt_{i+1}{}^{k-1} & \left(k-1\right)t_{i+1}{}^{k-2} & \cdots & 1 & 0\\ \vdots & \vdots & \cdots & \vdots & \vdots \\ k!t_{i+1} & \left(k-1\right)! & \cdots & 0 & 0 \end{array}\right]\left[\begin{array}{c} a_{j,k}^{i}\\ a_{j,k-1}^{i}\\ \vdots \\ a_{j,1}^{i}\\ a_{j,0}^{i} \end{array}\right]=\left[\begin{array}{c} B_{j,k}^{i}(t_{i})\\ B_{j,k}^{i}(t_{i+1})\\ B_{j,k}^{i}{}^{\left(1\right)}(t_{i+1})\\ \vdots \\ B_{j,k}^{i}{}^{\left(k-1\right)}(t_{i+1}) \end{array}\right]$$

The derivative of Equation (15) can be written as follows:

(18) $$\frac{d}{du}B_{i,k}(t)=B^{\prime }{}_{i,k}(t)=\frac{k}{t_{i+k}-t_{i}}B_{i,k-1}(t)-\frac{k}{t_{i+k+1}-t_{i+1}}B_{i+1,k-1}(t)$$

An arbitrary order derivative formula can be obtained as Equation (19) shows:

(19) $$B_{i,k}^{\left(d\right)}(t)=k(\frac{B_{i,k-1}^{\left(d-1\right)}(t)}{t_{i+k}-t_{i}}-\frac{B_{i+1,k-1}^{\left(d-1\right)}(t)}{t_{i+k+1}-t_{i+1}})$$

According to Equations (14), (15), (18) and (19), the coefficients $a_{j,k}^{i},a_{j,k-1}^{i},\cdots ,a_{j,1}^{i},a_{j,0}^{i}$ in Equation (16) can be obtained. Then the jth B-Spline basis curve $B_{j,k}^{i}\left(t\right)$ with degree *k* on vector $[t_{i},t_{i+1})$ can be calculated.

The B-Spline basis function $B_{j,k}\left(t\right)$ with degree *k* can also be represented in matrix form in the interval $\left[t_{0},t_{m}\right]$,

(20) $$\begin{array}{ll} B_{j,k}(t) & =\left[\begin{array}{cccc} B_{j,k}^{0}(t) & B_{j,k}^{1}(t) & \cdots & B_{j,k}^{m-1}(t) \end{array}\right]\\ & =\left[t^{k},t^{k-1},\cdots ,1\right]\left[\begin{array}{cccc} a_{j,k}^{0} & a_{j,k}^{1} & \cdots & a_{j,k}^{m-1}\\ \vdots & \vdots & \cdots & \vdots \\ a_{j,1}^{0} & a_{j,1}^{1} & \cdots & a_{j,1}^{m-1}\\ a_{j,0}^{0} & a_{j,0}^{1} & \cdots & a_{j,0}^{m-1} \end{array}\right]\\ & =v_{t}M_{e} \end{array}$$

The expression of different B-spline basis functions in an interval $[t_{i},t_{i+1})$ can be written as follows:

(21) $$\begin{array}{ll} B_{k}^{i}(t) & =\left[\begin{array}{cccc} B_{0,k}^{i}(t) & B_{1,k}^{i}(t) & \cdots & B_{n,k}^{i}(t) \end{array}\right]\\ & =\left[t^{k}\;t^{k-1}\;\cdots \;1\right]\left[\begin{array}{cccc} a_{0,k}^{i} & a_{1,k}^{i} & \cdots & a_{n,k}^{i}\\ \vdots & \vdots & \cdots & \vdots \\ a_{0,1}^{i} & a_{1,1}^{i} & \cdots & a_{n,1}^{i}\\ a_{0,0}^{i} & a_{1,0}^{1} & \cdots & a_{n,0}^{i} \end{array}\right]\\ & =v_{t}M_{e}\prime \end{array}$$

The coefficient matrixes $M_{e}$ in Equation (20) and $M_{e}^{\prime }$ in Equation (21) can be obtained by Equations (14), (15), (18) and (19).

The B-spline curve is a linear combination of control points $P_{i}$ and a B-spline basis function $B_{i,k}\left(t\right)$. The definition is as follows:

### 3.2. B-Spline Curve Definitions

**Definition** **2** [39]**.** *Given* n+1 *control points* $P_{i}$*, a knot vector* $T=\left\{t_{0}\leq t_{1}\leq \cdots \leq t_{m}\right\}$ *and B-spline basis functions* $B_{i,k}\left(t\right)$*, a B-spline curve is given by:*

(22) $$C\left(t\right)=\sum_{i=0}^{n}B_{i,k}\left(t\right)P_{i}$$

**Remark** **2.** *Indexes* m,n,k *must satisfy* m=n+k+1.

**Figure 3:**
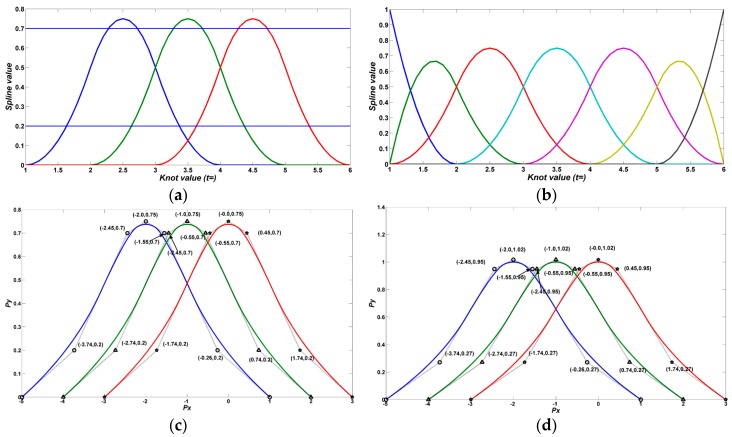
(**a**) Order 3 B-spline basis function in which the internal knot vector is $\left[\begin{array}{cc} 1 & \begin{array}{cc} 2 & \begin{array}{cc} 3 & \begin{array}{cc} 4 & \begin{array}{cc} 5 & 6 \end{array} \end{array} \end{array} \end{array} \end{array}\right]$; (**b**) Order 3 B-spline basis function that has multiple knots in which the internal knot vector is $\left[\begin{array}{ccc} \begin{array}{ccc} 1 & 1 & \begin{array}{cc} 1 & 2 \end{array} \end{array} & \begin{array}{ccc} 3 & 4 & 5 \end{array} & \begin{array}{ccc} 6 & 6 & 6 \end{array} \end{array}\right]$; (**c**) Order 3 B-spline functions with multiple knots; the internal knot vector is $\left[\begin{array}{ccc} \begin{array}{ccc} 1 & 1 & \begin{array}{cc} 1 & 2 \end{array} \end{array} & \begin{array}{ccc} 3 & 4 & 5 \end{array} & \begin{array}{ccc} 6 & 6 & 6 \end{array} \end{array}\right]$; (**d**) Amplitude standardization of c; the internal knot vector is $\left[\begin{array}{ccc} \begin{array}{ccc} 1 & 1 & \begin{array}{cc} 1 & 2 \end{array} \end{array} & \begin{array}{ccc} 3 & 4 & 5 \end{array} & \begin{array}{ccc} 6 & 6 & 6 \end{array} \end{array}\right]$.

**Remark** **3.** *If the first knot and the last knot have multiplicity with value k* + 1*, the B-spline curve is called closed clamped; otherwise, it is called an open clamped B-spline curve. More specific illustrations can be seen in Figure 3.*

From Equation (22) on the ith vector the B-spline function can be written as:

(23) $$\begin{array}{ll} C_{i}(t) & =\left[B_{0,k}^{i}\;B_{1,k}^{i}\;\cdots \;B_{n,k}^{i}\right]{\left[P_{0}\;P_{1}\;\cdots \;P_{n}\right]}^{T}\\ & =v_{t}M_{e}\prime {\left[P_{0}\;P_{1}\;\cdots \;P_{n}\right]}^{T}\\ & =v_{t}M_{e}\prime {\left[\left(Px_{0},Py_{0})\;(Px_{1},Py_{1})\;\cdots \;(Px_{n},Py_{n}\right)\right]}^{T} \end{array}$$

If the horizontal value $x_{in}$ of an arbitrary point $\left(x_{in},y_{in}\right)$ on the B-spline curve is known, the following formula can be obtained by:

(24) $$x_{in}=v_{t}M_{e}\prime {\left[Px_{0}\;Px_{1}\;\cdots \;Px_{n}\right]}^{T}$$

Therefore, the unknown internal vector $v_{\hat{t}}$ can be calculated. Furthermore, the vertical value $y_{in}$ can be obtained:

(25) $$y_{in}=v_{\hat{t}}M_{e}\prime {\left[Py_{0}\;Py_{1}\;\cdots \;Py_{n}\right]}^{T}$$

In this paper, order 3 B-spline curve is adopted. Then Equation (23) can be simplified to the following form:

(26) $$\begin{array}{ll} C_{j}(u) & =\left[t^{2}\;t^{1}\;1\right]\left[\begin{array}{ccc} a_{2}^{1} & a_{2}^{2} & a_{2}^{3}\\ a_{1}^{1} & a_{1}^{2} & a_{1}^{3}\\ a_{0}^{1} & a_{0}^{2} & a_{0}^{3} \end{array}\right]{\left[P_{j}\;P_{j+1}\;P_{j+2}\right]}^{T}\\ & =\left[t^{2}\;t^{1}\;1\right]M_{e}\prime {\left[P_{j}\;P_{j+1}\;P_{j+2}\right]}^{T} \end{array}$$

$x_{in}$ can be represented as $x_{in}=\left[\begin{array}{ccc} t^{2} & t & 1 \end{array}\right]A\beta$, in which $M_{e}^{\prime }={\left(\alpha_{1},\alpha_{2},\alpha_{3}\right)}^{T}$, $\beta ={\left[\begin{array}{ccc} Px_{0} & Px_{1} & Px_{2} \end{array}\right]}^{T}$. According to the known horizontal coordinate value $x_{in}$, the corresponding internal knot $\hat{t}$ can be obtained:

(27) $$\hat{t}=\frac{-\left(\alpha_{2}\cdot \beta \right)\pm \sqrt{{\left(\alpha_{2}\cdot \beta \right)}^{2}-4\left(\alpha_{1}\cdot \beta \right)\left[\left(\alpha_{3}\cdot \beta \right)-x_{in}\right]}}{2\left(\alpha_{1}\cdot \beta \right)},\left(\alpha_{1}\cdot \beta \right)\neq 0$$

Furthermore:

(28) $$\begin{array}{ll} y_{in} & =\left[\begin{array}{ccc} {\hat{t}}^{2} & \hat{t} & 1 \end{array}\right]{\left[\begin{array}{ccc} \left(\alpha_{1}\cdot \gamma \right) & \left(\alpha_{2}\cdot \gamma \right) & \left(\alpha_{3}\cdot \gamma \right) \end{array}\right]}^{T}\\ & =(\alpha_{1}\cdot \gamma ){\hat{t}}^{2}+(\alpha_{2}\cdot \gamma )\hat{t}+(\alpha_{3}\cdot \gamma ) \end{array}$$

in which $\gamma ={\left[\begin{array}{ccc} Py_{0} & Py_{1} & Py_{2} \end{array}\right]}^{T}$.

According to the Definition 2, the B-spline function is improved in this paper by proposing two deformation factors called translation factor and scaling factor.

**Definition** **3.** *Given* n+1 *control points* $P_{i}$ *and a knot vector* $T=\left\{t_{0}\leq t_{1}\leq \cdots \leq t_{m}\right\}$*, a new B-spline function of degree k can be written as:*

(29) $$\begin{array}{ll} C\prime (t) & =\sum_{i=0}^{n}P_{i}\prime B_{i,k}(t)\\ & =\sum_{i=0}^{n}\left(Px_{i}+\lambda t_{i}+\kappa w_{i},Py_{i})B_{i,k}(t\right)\\ & =\sum_{i=0}^{n}\left[P_{i}+(\lambda b_{i},0)+(\kappa w_{i},0)B_{i,k}(t)\right] \end{array}$$

**Remark** **4.** *At the beginning, the value of the two deformation factors* λ *and* κ *are zero. thus, the formulas in Definition 2 and 3 are equivalent.* λ *is called translation factor, and* κ *is called scaling factor.*

**Assumption** **1.** *The two factors specify how the shape changes, assuming that* $b={\left[\begin{array}{cc} 1 & \begin{array}{ccc} 1 & \cdots & 1 \end{array} \end{array}\right]}_{1\times n}$, $w={\left[\begin{array}{ccc} \underset{\rho }{\underset{︸}{\begin{array}{ccc} 1 & 1 & 1 \end{array}}} & \underset{\tau }{\underset{︸}{0}} & \underset{\rho }{\underset{︸}{\begin{array}{ccc} -1 & -1 & -1 \end{array}}} \end{array}\right]}_{1\times n}$ *and* 2×ρ+τ=n *(*τ *can be 0 or 1).*

### 3.3. The B-Spline Neural Network

**Figure 4:**
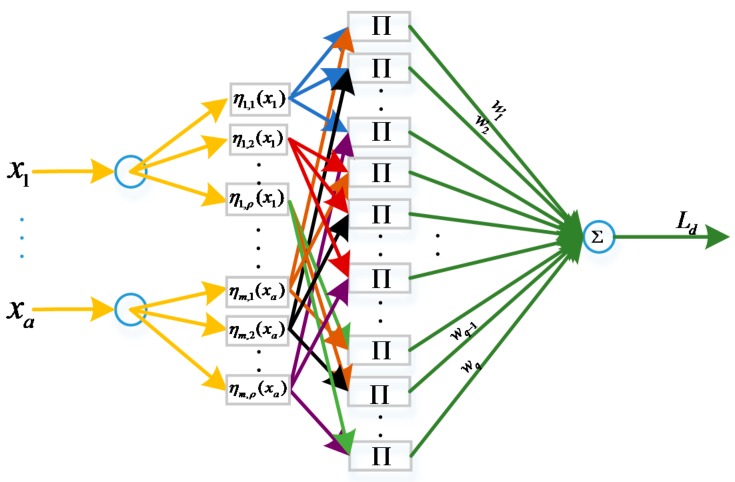
The general structure of BSNN.

Compared with some previous studies, the proposed B-spline adopts an order 3 B-spline function as activation function rather than a B-spline basis function as in some references [29,31]. Generally speaking, neural networks always have more than one hidden layer. The structure of our BSNN is shown in Figure 4.

As shown in Figure 4, the neural network system has multiple inputs and one output. Each input corresponds to several activation functions. The output of BSNN can be mapped from inputs $x_{i}$ to an output $L_{d}$ by using B-splines as activation functions. The output of the hidden layer can be written as follows:

(30) $$h_{l}=\prod_{i=1}^{a}\eta_{i,j}\left(x_{i}\right),j=1,2,\cdots ,\rho$$

In which, $\eta_{i,j}\left(x_{i}\right)$ means the ith input corresponding to the jth activation function; ρ means the number of inputs; a means the number of activation functions. Therefore, the output of BSNN is as follows:

(31) $$L_{d}=\sum_{l=1}^{L}\omega_{l}h_{l}=\sum_{l=1}^{L}\omega_{l}\left(\prod_{i=1}^{\rho }\eta_{i,j}\left(x_{i}\right)\right),L=a^{\rho }$$

The training method is to minimize the error function which is defined as follows:

(32) E(t)=V

The chain rules of parameter update can be written as follows:

(33) $$\frac{\partial E}{\partial s}\frac{\partial s}{\partial l_{d}}=\frac{p+1}{\beta }{\left\|\dot{e}\right\|}^{p}\left\|s\right\|$$

(34) $$\Delta \omega_{i}=-\frac{\partial E}{\partial \omega_{i}}=-\frac{\partial E}{\partial s}\frac{\partial s}{\partial l_{d}}\frac{\partial l_{d}}{\partial \omega_{i}}=\frac{p+1}{\beta }{\left\|\dot{e}\right\|}^{p}\left\|s\right\|h_{l}$$

(35) $$\omega_{i}\left(t\right)=\omega_{i}\left(t-1\right)+\eta \Delta \omega_{i}+\alpha [\omega_{i}(t-1)-\omega_{i}(t-2)]$$

(36) $$\Delta \lambda_{i,j}=-\frac{\partial E}{\partial \lambda_{i,j}}=-\frac{\partial E}{\partial s}\frac{\partial s}{\partial l_{d}}\frac{\partial l_{d}}{\partial h_{l}}\frac{\partial h_{l}}{\partial \eta_{i,j}}\frac{\partial \eta_{i,j}}{\partial \lambda_{i,j}}$$

(37) $$\lambda_{i,j}\left(t\right)=\lambda_{i,j}\left(t-1\right)+\eta \Delta \lambda_{i,j}+\alpha [\lambda_{i,j}\left(t-1\right)-\lambda_{i,j}\left(t-2\right)]$$

(38) $$\Delta \kappa_{i,j}=-\frac{\partial E}{\partial \kappa_{i,j}}=-\frac{\partial E}{\partial s}\frac{\partial s}{\partial l_{d}}\frac{\partial l_{d}}{\partial h_{l}}\frac{\partial h_{l}}{\partial \eta_{i,j}}\frac{\partial \eta_{i,j}}{\partial \kappa_{i,j}}$$

(39) $$\kappa_{i,j}\left(t\right)=\kappa_{i,j}\left(t-1\right)+\eta \Delta \kappa_{i,j}+\alpha [\kappa_{i,j}\left(t-1\right)-\kappa_{i,j}\left(t-2\right)]$$

(40) $$\frac{\partial l_{d}}{\partial h_{l}}\frac{\partial h_{l}}{\partial \eta_{i,j}}=w_{l}\prod_{\begin{array}{l} s=1\\ s\neq i \end{array}}^{\rho -1}\eta_{s,j}(x_{s}),j=1,2,\cdots a$$

In these equations, η is the learning rate; α is the inertial coefficient.

(41) $$\begin{array}{ll} \frac{\partial \eta_{i,j}(x_{j})}{\partial \lambda_{i,j}} & =\frac{\partial [\sum_{s=0}^{n}(P_{s}^{{}_{i,j}}+\lambda_{i,j}t_{s}+\kappa_{i,j}b_{s})B_{s,k}\left(\hat{t}\right)]}{\partial \lambda_{i,j}}\\ & =\frac{\partial [\sum_{s=0}^{n}P_{s}^{{}_{i,j}}B_{s,k}\left(\hat{t}\right)+\sum_{s=1}^{n}\lambda_{i,j}t_{s}B_{s,k}\left(\hat{t}\right)+\sum_{s=1}^{n}\kappa_{i,j}b_{s}B_{s,k}\left(\hat{t}\right)]}{\partial \lambda_{i,j}}\\ & =\sum_{s=0}^{n}t_{s}B_{s,k}\left(\hat{t}\right) \end{array}$$

The B-spline basis function has an important property which is called “Partition of Unity”, indicating that the sum of all non-zero degree *k* basis functions on span $[t_{i},t_{i+1})$ is 1, i.e., $\sum_{s=0}^{n}B_{s,k}\left(t\right)=1$.

According to Assumption 1, the following Equations (42) and (44) can be obtained:

(42) $$\frac{\partial \eta_{i,j}(x_{j})}{\partial \lambda_{i,j}}=\sum_{s=0}^{n}B_{s,k}\left(\hat{t}\right)=1$$

(43) $$\begin{array}{ll} \frac{\partial \eta_{i,j}(x_{j})}{\partial \kappa_{i,j}} & =\frac{\partial [\sum_{s=0}^{n}(P_{s}^{i,j}+\lambda_{i,j}t_{s}+\kappa_{i,j}w_{s})B_{s,k}\left(\hat{t}\right)]}{\partial \kappa_{i,j}}\\ & =\frac{\partial [\sum_{s=0}^{n}P_{i,j}B_{s,k}\left(\hat{t}\right)+\lambda_{i,j}t_{s}B_{s,k}\left(\hat{t}\right)+\kappa_{i,j}w_{s}B_{s,k}\left(\hat{t}\right)]}{\partial \kappa_{i,j}}\\ & =\sum_{s=0}^{n}w_{s}B_{s,k}\left(\hat{t}\right) \end{array}$$

(44) ∂ηi,j(xj)∂κi,j={∑s=0(n−1)2Bs,k(t^)−∑s=(n+1)/2nBs,k(t^), n is odd∑s=0(n−2)2Bs,k(t^)−∑s=(n+2)/2nBs,k(t^),n is even

Therefore, an adaptive BSNN is proposed. Experiments and simulations carried out to verify the performance of the BSNN are discussed in the following section.

## 4. Results of Experiment and Simulation

**Figure 5:**
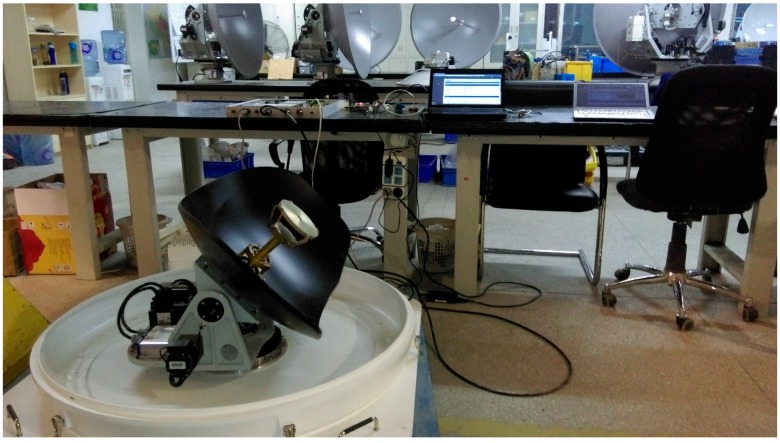
Experimental two axis MSA system.

Experiments and simulations were carried out to validate the performance of the proposed BSNN-based NFTSM control method in this section. The experimental system is shown in Figure 5. Comparisons are made between the results of the conventional PID method, the TSM in reference [19], the NTSM in reference [15], the NFTSM in this paper, the RBF neural network-based NFTSM. The parameters of the MSA control system are listed in Table 1.

In this system, the BSNN has two inputs and one output. Each of the inputs corresponds to three B-spline activation functions which have n + 1 = 7 control points separately. The internal knot vector of B-Spline curve is $\left[\begin{array}{ccc} \begin{array}{ccc} 1 & 1 & \begin{array}{cc} 1 & 2 \end{array} \end{array} & \begin{array}{ccc} 3 & 4 & 5 \end{array} & \begin{array}{ccc} 6 & 6 & 6 \end{array} \end{array}\right]$ and the degree of B-spline curve is 2. Moreover, the learning rate η=0.6, the inertial coefficient α=0.05 and c=5. $l_{d}=7$ is adopted for NFTSM control method.

### 4.1. Description of the Coupling Effect

**Figure 6:**
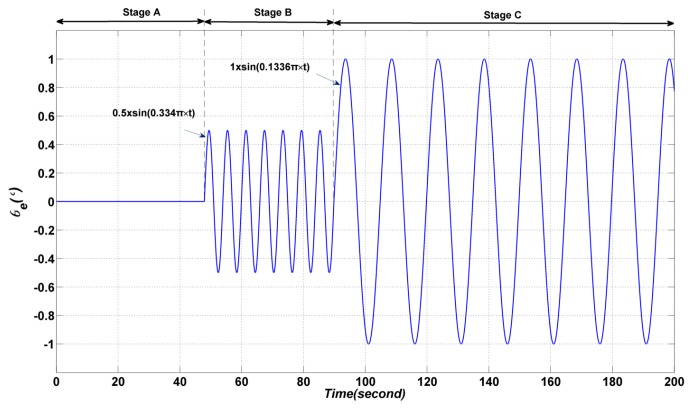
The sinusoidal motion of the elevation gimbal.

**Figure 7:**
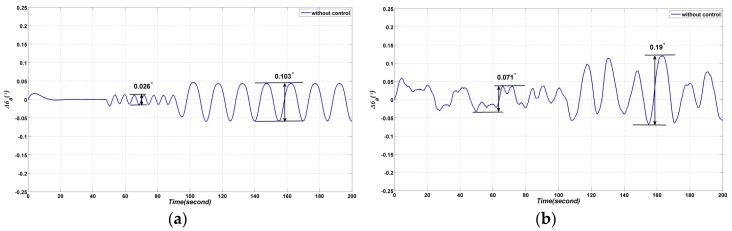
The coupling effect of the azimuth gimbal: (**a**) simulated coupling effect result; (**b**) experimental coupling effect result.

In this section, the amplitude of the elevation gimbal is 0 degrees in the first 47.9 s (Stage A); the gimbal is assigned to perform a sinusoidal motion 0.5×sin(0.334×π×t) from 47.9 s to 89.8 s (Stage B); the gimbal is assigned to perform a sinusoidal motion 1.0×sin(0.1336×π×t) from 89.8 s to 200 s (Stage C), as shown in Figure 6. In order to describe the coupling effect caused by the elevation and vehicle movement, there is no control and no input applied to the azimuth gimbal. The vehicle is assigned to move with speed of 0.5°/s. Simulation and experiment results are demonstrated in Figure 7.

As shown in Figure 7, the azimuth gimbal has a large angular output in both the simulation and experiment. The azimuth gradually increases due to the motion of the elevation gimbal. Apparently, the results are not what the controller engineer expected. Therefore, a decoupling controller is required.

### 4.2. Description of De-Coupling Effect

**Figure 8:**
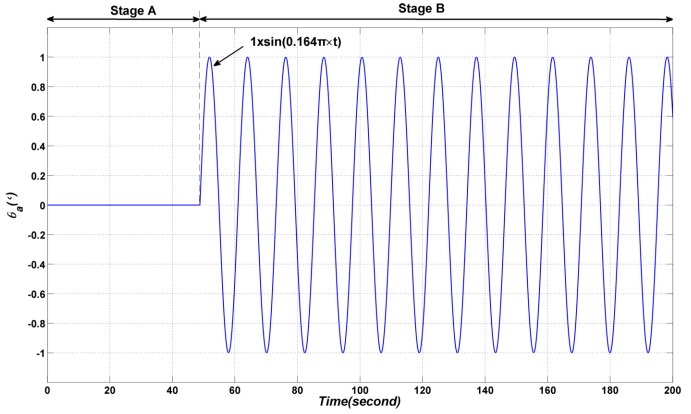
The sinusoidal motion of the azimuth gimbal.

In order to validate the decoupling ability of the proposed method, the azimuth gimbal is assigned to perform a sinusoidal motion with amplitude 0 in the first 48.8 s (Stage A); a sinusoidal motion 1.0×sin(0.164×π×t) is applied in the following period (Stage B), as shown in Figure 8. The elevation gimbal is still assigned to perform a sinusoidal motion as in Section 4.1.

#### 4.2.1. Simulation Results

**Figure 9:**
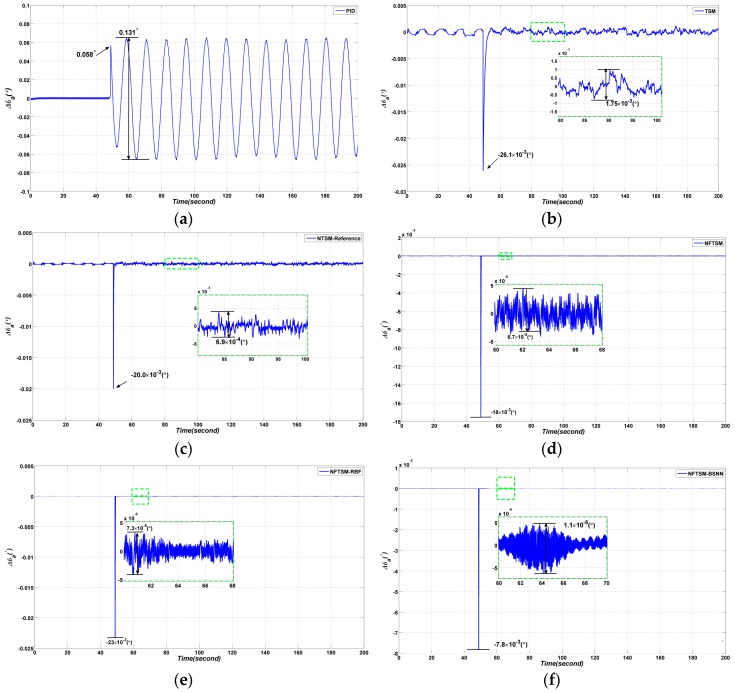
Comparative azimuth gimbal absolute angle simulation results with four different methods: (**a**) PID method; (**b**) TSM method in reference [19]; (**c**) NTSM method in reference [15]; (**d**) the proposed NFTSM method; (**e**) NFTSM-RBFNN method; (**f**) the proposed NFTSM-BSNN method.

The simulation results are shown in Figure 9.

From the simulation results, the following conclusions can be obtained: the conventional PID method has the worst performance, the peak to peak (*p*–*p*) value of tracking error is $0.131^{{}^{\circ}}$*,* the tracking error at time 48.8 s is $0.058^{{}^{\circ}}$; while the tracking error $\Delta \theta_{a}$ of the conventional TSM method is $1.75\times 10^{-3}\left({}^{\circ}\right)$ and the tracking error at time 48.8 s is $-26.1\times 10^{-3}\left({}^{\circ}\right)$; the tracking error $\Delta \theta_{a}$ of the NTSM method in reference is $6.9\times 10^{-4}\left({}^{\circ}\right)$, the tracking error is $-20.0\times 10^{-3}\left({}^{\circ}\right)$; the tracking error $\Delta \theta_{a}$ of NFTSM is $6.7\times 10^{-5}\left({}^{\circ}\right)$, and the tracking error at time 48.8 s is $18.0\times 10^{-3}\left({}^{\circ}\right)$; for RBFNN based NFTSM the tracking error $\Delta \theta_{a}$ is $7.3\times 10^{-5}\left({}^{\circ}\right)$ and the tracking error at time 48.8 s is $23.0\times 10^{-3}\left({}^{\circ}\right)$; the best performance corresponds to the BSNN-based NFTSM with a tracking error of $1.1\times 10^{-5}\left({}^{\circ}\right)$ and the tracking error at time 48.8 s of $7.8\times 10^{-3}\left({}^{\circ}\right)$. At time 48.8 s, the azimuth gimbal begins to perform the assigned sinusoidal motion from a still state. The simulation results show that the proposed BSNN has a better adaptive capability and the proposed controller shows better decoupling effect than other controllers.

**Figure 10:**
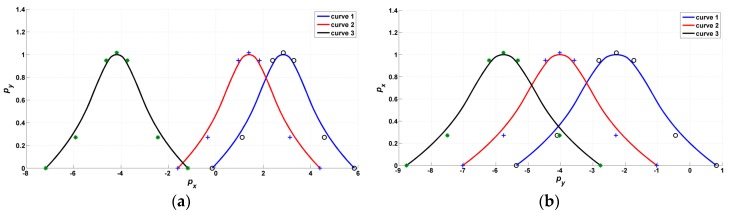
Form change of the B-spline activation function: (**a**) initial forms of the activation curves; (**b**) final forms of the activation curves after training.

The forms of the B-spline curves are shown in Figure 10. It can be seen from the figure that both the positions and the shapes of the B-spline curves are changed.

#### 4.2.2. Experiment Results

**Figure 11:**
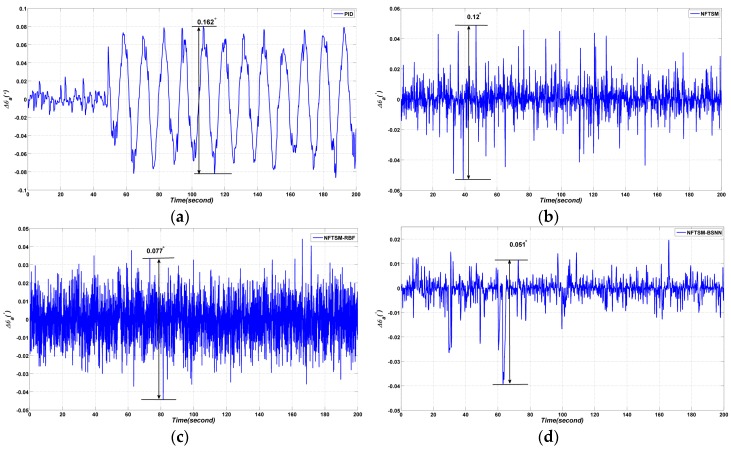
Comparative azimuth gimbal absolute angle experimental results with four different methods: (**a**) PID method; (**b**) NFTSM method; (**c**) NFTSM-RBFNN method; (**d**) the proposed method.

Decoupling effect experiments are carried out in this section. The results are shown in Figure 11. As shown in Figure 11, the proposed BSNN-based NFTSM performs better than the other three methods. The *p*–*p* value of $\Delta \theta_{a}$ is $0.162^{{}^{\circ}}$ when the PID controller is applied; the *p*–*p* value of $\Delta \theta_{a}$ is $0.12^{{}^{\circ}}$ when the NFTSM controller is applied; the *p*–*p* value of $\Delta \theta_{a}$ is $0.077^{{}^{\circ}}$ when the RBFNN- based NFTSM is applied; the *p*–*p* value of $\Delta \theta_{a}$ is $0.051^{{}^{\circ}}$ when the BSNN-based NFTSM is applied.

### 4.3. Disturbance Rejecting Ability Simulations and Experimental Results

**Figure 12:**
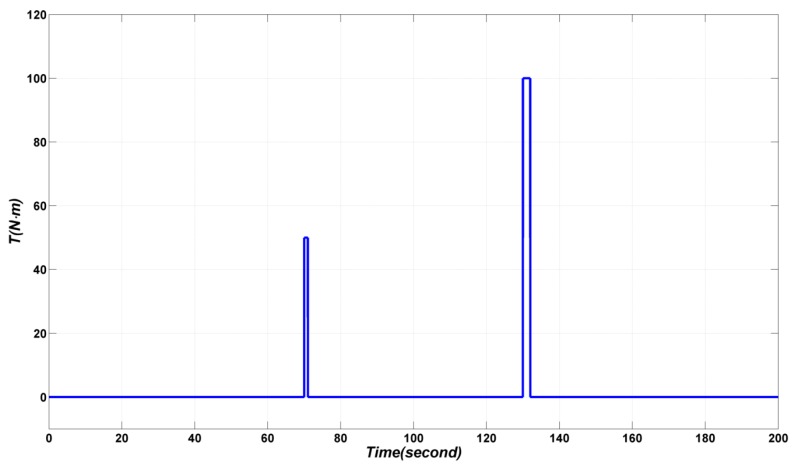
Pulse disturbances added to the system.

In order to validate the disturbance rejecting ability of the proposed method, pulse disturbances of 50 N·m and 100 N·m are added at time 70 s and 130 s, respectively, as shown in Figure 12.

#### 4.3.1. Simulation Results

**Figure 13:**
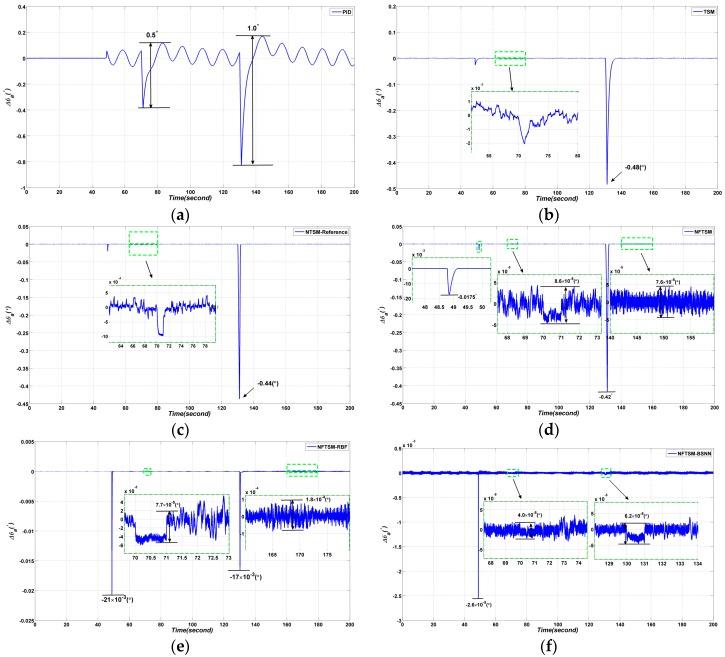
Comparative azimuth gimbal absolute angle simulation results with four different methods: (**a**) PID method; (**b**) TSM method in reference [19]; (**c**) NTSM method in reference [15]; (**d**) the proposed NFTSM method; (**e**) NFTSM-RBFNN method; (**f**) the proposed NFTSM-BSNN method.

The disturbance rejecting ability simulation results are shown in Figure 13. From the simulation results in Figure 13, the *p*–*p* values of $\Delta \theta_{a}$ are $0.5^{{}^{\circ}}$ and $1.0^{{}^{\circ}}$ when the disturbances are added to the conventional PID controller at time 70 s and 130 s, respectively, while the tracking errors of the conventional TSM method are $-2.0\times 10^{-3}\left({}^{\circ}\right)$ and $-0.48^{{}^{\circ}}$ at time 70 s and 130 s, respectively; the tracking errors $\Delta \theta_{a}$ of the NTSM method in reference are $9.9\times 10^{-4}\left({}^{\circ}\right)$ and $-0.44^{{}^{\circ}}$; the *p*–*p* values of $\Delta \theta_{a}$ are $8.6\times 10^{-5}\left({}^{\circ}\right)$ and $0.42^{{}^{\circ}}$ at time 70 s and 130 s when NFTSM is applied, respectively.

**Figure 14:**
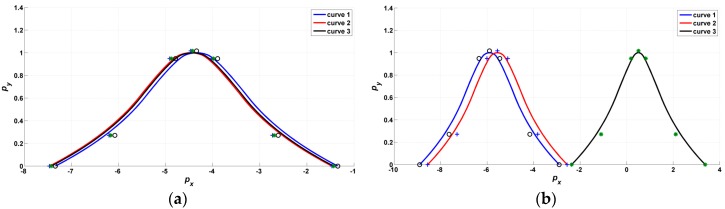
Form change of the B-spline activation function: (**a**) initial forms of the curves; (**b**) final forms of the curves after training.

However, when the RBFNN-based NFTSM is applied, the *p*–*p* values of $\Delta \theta_{a}$ are $7.7\times 10^{-5}\left({}^{\circ}\right)$ and $17\times 10^{-3}\left({}^{\circ}\right)$ at time 70 s and 130 s, respectively; the *p*–*p* values of $\Delta \theta_{a}$ are $4.0\times 10^{-5}\left({}^{\circ}\right)$ and $6.2\times 10^{-5}\left({}^{\circ}\right)$ at time 70 s and 130 when the BSNN-based NFTSM is applied, respectively. Therefore, it is apparent that the neural network is good at nonlinear approximation, and the proposed BSNN performs better. The forms of B-spline curves are shown in Figure 14.

#### 4.3.2. Experimental Results

**Figure 15:**
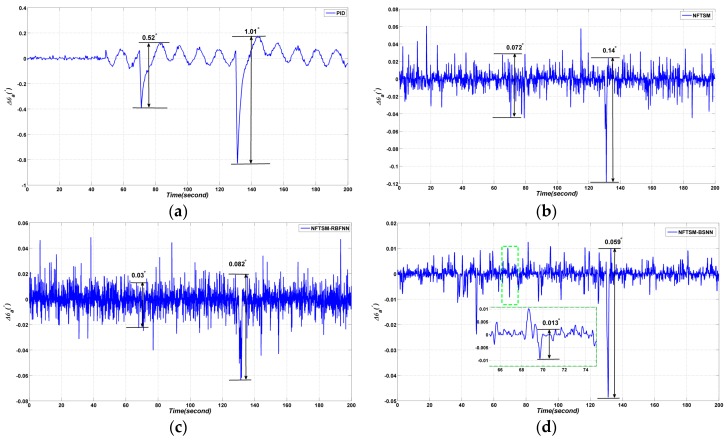
Comparative azimuth gimbal absolute angle experimental results with four different methods: (**a**) PID method; (**b**) NFTSM method; (**c**) NFTSM-RBFNN method; (**d**) the proposed method.

Four different methods are applied to the control system of MSA. From the experimental results in Figure 15, the following conclusions can be obtained: the *p*–*p* values of $\Delta \theta_{a}$ are $0.52^{{}^{\circ}}$ and $1.01^{{}^{\circ}}$ when the disturbances are added to PID controller at time 70 s and 130 s, respectively; whereas the tracking values for NFTSM are $0.072^{{}^{\circ}}$ and $0.14^{{}^{\circ}}$ at time 70 s and 130 s, respectively; the tracking values for RBFNN based NFTSM are $0.03^{{}^{\circ}}$ and $0.082^{{}^{\circ}}$ at time 70 s and 130 s, respectively; when BSNN based NFTSM is applied, the *p*–*p* values of are $0.013^{{}^{\circ}}$ at time 70 s and $0.059^{{}^{\circ}}$ at time 130 s.

According to the simulations and experiments, it can be seen that the proposed BSNN based NFTSM is good at nonlinear approximate and has strong self-adaptability.

## 5. Conclusions

The paper focuses on the inertial sensor-based two gimbal mobile satcom antenna. In order to obtain high quality communications, the MSA should point to the specific satellite when the carrier is moving. The dynamic model of MSA is established based on traditional Newton-Euler method and the corresponding control block diagram is built. In this paper, the non-singular fast terminal sliding mode control is adopted and developed to increase the line of sight stabilization accuracy. Meanwhile, the features of existence and convergence in finite time are proved. Then a neural network is employed to approximate the nonlinear item in the system. In addition, a novel BSNN is proposed and used in this paper. A brief study of B-spline basis and B-spline function is also carried out, then the computational function used to obtain the arbitrary point on the curve is derived. The B-spline function is reformed to enhance its adaptive capacity. To validate the effectiveness of the proposed NFTSM and BSNN, simulations and experiments are conducted. Results of different methods, including PID, NFTSM, NFTSM-RBF, NFTSM-BSNN, are compared in this paper. It is shown that the proposed method has better decoupling effects and disturbance rejecting ability than the others. Because the B-spline curve has an excellent ability called “local control”, it can be used to approximate arbitrarily shaped curves. According to the analysis in this paper, we can conclude that the proposed BSNN is good at nonlinear approximation owing to its local features. The robustness of the system can also be improved by applying this method.

## Figures and Tables

**Table 1:** Parameters of MSA system.

Parameters	Values	Unit	Parameters	Values	Unit	Parameters	Values	Unit
$J_{\mathrm{ex}}$	0.51	$\mathrm{kg}\cdot m^{2}$	$J_{\mathrm{ey}}$	0.54	$\mathrm{kg}\cdot m^{2}$	$J_{\mathrm{ez}}$	0.59	$\mathrm{kg}\cdot m^{2}$
$J_{\mathrm{ax}}$	1.49	$\mathrm{kg}\cdot m^{2}$	$J_{\mathrm{ay}}$	1.56	$\mathrm{kg}\cdot m^{2}$	$J_{\mathrm{az}}$	1.42	$\mathrm{kg}\cdot m^{2}$
$J_{\mathrm{ad}}$	0.96	$\mathrm{kg}\cdot m^{2}$	$J_{\mathrm{af}}$	1.37	$\mathrm{kg}\cdot m^{2}$	$N_{a}$	2.8	N·m/A
$k_{\mathrm{at}}$	0.42		$k_{\mathrm{ab}}$	0.42		$R_{\mathrm{aa}}$	1.23	Ω
$f_{\mathrm{ad}}$	0.3		$f_{\mathrm{af}}$	0.5				

## Appendix A

The Terminal sliding mode surface (8) can be written as follows:

(A1) $$s=\left\|\hat{e}\right\|sign\left(\hat{e}\right)+\frac{1}{\alpha }{\left\|\hat{e}\right\|}^{r+1}sign\left(\hat{e}\right)+\frac{1}{\beta }{\left\|\dot{\hat{e}}\right\|}^{p+1}sign\left(\dot{\hat{e}}\right)=0$$

The following formula can be obtained:

(A2) $$sign\left(\hat{e}\right)=-sign\left(\dot{\hat{e}}\right)$$

Then Equation (A1) can be further simplified as:

(A3) $$\left\|\hat{e}\right\|+\frac{1}{\alpha }{\left\|\hat{e}\right\|}^{r+1}=\frac{1}{\beta }{\left\|\dot{\hat{e}}\right\|}^{p+1}$$

It is apparent that:

(A4) (β‖e^‖+βα‖e^‖r+1)1p+1=‖e^˙‖=e^˙sign(e^˙)

Then the following formula can be obtained:

(A5) −(β‖e^‖+βα‖e^‖r+1)1p+1sign(e^)=e^˙

If $\hat{e}\geq 0$,

(A6) −(βe^+βαe^r+1)1p+1=e^˙

Else if $\hat{e}<0$,

(A7) (β(−e^)+βα(−e^)r+1)1p+1=e^˙

Equation (A5) can be integrated and written as follows:

(A8) ∫0e^(t0)d(−e^)(β(−e^)+βα(−e^)r+1)1p+1+∫0e^(t0)de^(βe^+βαe^r+1)1p+1=∫0tdt

(A9) ∫0|e^(t0)|de^(βe^)1p+1≥∫0tdt

Then the convergence time can be obtained:

(A10) t≤p+1pβ1p+1|e^(t0)|pp+1

This ends the proof.

## References

[1] Densmore A.C., Jamnejad V. (1993). A satellite-tracking k- and ka-band mobile vehicle antenna system. IEEE Trans. Veh. Technol..

[2] Cheng W., Minzhe T., Weizhou S. An h2/h_∞_ control design for mobile satcom antenna servo systems. Proceedings of the 2016 35th Chinese Control Conference (CCC).

[3] Marsh E.A. (2008). Inertially Stabilized Platforms for Satcom on-the-Move Applications: A Hybrid Open/Closed-Loop Antenna Pointing Strategy. Master’s degree.

[4] Debruin J. (2008). Control systems for mobile satcom antennas. IEEE Control Syst..

[5] Tian F., Wang R., Gao F., Yao M. (2014). A beam stabilization algorithm based on nonlinear observer for low cost satcom-on-the-move. China Commun..

[6] Rue A.K. (1969). Stabilization of precision electrooptical pointing and tracking systems. IEEE Trans. Aerosp. Electr. Syst..

[7] Shuang C., Ke D., Shang W. (2011). Modeling analysis on the gyro stabilized. Sci. Technol. Rev..

[8] Fang J., Yin R., Lei X. (2015). An adaptive decoupling control for three-axis gyro stabilized platform based on neural networks. Mechatronics.

[9] Ito K., Maebashi W., Ikeda J., Iwasaki M. Fast and precise positioning of rotary table systems by feedforward disturbance compensation considering interference force. Proceedings of the IECON 2011 37th Annual Conference on IEEE Industrial Electronics Society.

[10] Řezáč M., Hurák Z. Vibration rejection for inertially stabilized double gimbal platform using acceleration feedforward. Proceedings of the IEEE International Conference on Control Applications (CCA).

[11] Mu Q., Liu G., Lei X. (2014). A RBFNN-based adaptive disturbance compensation approach applied to magnetic suspension inertially stabilized platform. Math. Probl. Eng..

[12] Moorty J.A.R.K., Marathe R., Sule V.R. (2002). H_∞_ control law for line-of-sight stabilization for mobile land vehicles. Opt. Eng..

[13] Li S., Zhong M., Zhao Y. (2014). Estimation and compensation of unknown disturbance in three-axis gyro-stabilized camera mount. Trans. Inst. Meas. Control.

[14] Ma D., Lin H. Chattering-free nonsingular fast terminal sliding-mode control for permanent magnet synchronous motor servo system. Proceedings of the 35th Chinese Control Conference (CCC).

[15] Feng Y., Yu X., Man Z. (2002). Non-singular terminal sliding mode control of rigid manipulators. Automatica.

[16] Zak M. (1988). Terminal attractors for addressable memory in neural networks. Phys. Lett. A.

[17] Wu Y., Yu X., Man Z. (1998). Terminal sliding mode control design for uncertain dynamic systems. Syst. Control Lett..

[18] Madani T., Daachi B., Djouani K. (2016). Non-singular terminal sliding mode controller: Application to an actuated exoskeleton. Mechatronics.

[19] Yu S., Yu X., Stonier R. Continuous finite-time control for robotic manipulators with terminal sliding modes. Proceedings of the 6th International Conference of Information Fusion.

[20] Ding F., Cui Y., Wang C., Zhang X. Course control of air cushion vessel based on terminal sliding mode control with rbf neural network. Proceedings of the 35th Chinese Control Conference (CCC).

[21] Shtessel Y., Edwards C., Fridman L., Levant A. (2015). Sliding Mode Control and Observation.

[22] Levant A. (2001). Universal single-input-single-output (siso) sliding-mode controllers with finite-time convergence. IEEE Trans. Autom. Control.

[23] Aghababa M.P. (2013). Design of a chatter-free terminal sliding mode controller for nonlinear fractional-order dynamical systems. Int. J. Control.

[24] Tran M.D., Kang H.J. (2017). Adaptive terminal sliding mode control of uncertain robotic manipulators based on local approximation of a dynamic system. Neurocomputing.

[25] Jia T., Kang G. An rbf neural network-based nonsingular terminal sliding mode controller for robot manipulators. Proceedings of the 2012 Third International Conference on Intelligent Control and Information Processing (ICICIP).

[26] Wang H., Yang Z., Zhou Z. Rbf-based terminal sliding mode control for a class of underactuated mechanical system. Proceedings of the 2016 Chinese Control and Decision Conference (CCDC).

[27] Fang Y., Liu L., Li J., Xu Y. (2015). Decoupling control based on terminal sliding mode and wavelet network for the speed and tension system of reversible cold strip rolling mill. Int. J. Control.

[28] Lin C.K. (2006). Nonsingular terminal sliding mode control of robot manipulators using fuzzy wavelet networks. IEEE Trans. Fuzzy Syst..

[29] Ferch M., Zhang J., Knoll A. Robot skill transfer based on B-Spline fuzzy controllers for force-control tasks. Proceedings of the 1999 IEEE International Conference on Robotics and Automation.

[30] Yiu K.F., Wang S., Teo K.L., Tsoi A.C. (2001). Nonlinear system modeling via knot-optimizing B-Spline networks. IEEE Trans. Neural Netw..

[31] Cabrita C., Ruano A.E., Fonseca C.M. Single and multi-objective genetic programming design for B-Spline neural networks and Neuro-Fuzzy systems. Proceedings of the Ifac Workshop on Advanced Fuzzy-Neural Control.

[32] Leandro D.S.C., Guerra F.A. (2008). B-spline neural network design using improved differential evolution for identification of an experimental nonlinear process. Appl. Soft Comput..

[33] Qamar S., Khan L., Qamar Z. Online adaptive full car active suspension control using b-spline fuzzy-neural network. Proceedings of the 2013 11th International Conference on Frontiers of Information Technology (FIT).

[34] Qamar S., Khan L., Ali S. (2013). Adaptive B-Spline based Neuro-Fuzzy control for full car active suspension system. Middle East J. Sci. Res..

[35] Wang C.H., Wang W.Y., Lee T.T., Tseng P.S. (1995). Fuzzy B-Spline membership function and its applications in Fuzzy-Neural control. IEEE Trans. Syst. Man Cybern..

[36] Mar J., Lin F.J., Lin H.T., Hsu L.C. (2003). Evolutionary learning of BMF Fuzzy-Neural networks using a reduced-form genetic algorithm. IEEE Trans. Syst. Man Cybern. Part B Cybern..

[37] Mao J., Wang Y., Sun W. (2002). Remote sensing images classification using fuzzy B-Spline function neural network. J. Electr. Meas. Instrom..

[38] Coelho L.D.S., Pessôa M.W. (2009). Nonlinear identification using a B-Spline neural network and chaotic immune approaches. Mech. Syst. Signal Proc..

[39] Boor C.D. (1985). A practical Guide to Splines/Arl De Boor.

[40] B-Splines. http://web.mit.edu/hyperbook/Patrikalakis-Maekawa-Cho/node16.html.

